# Editorial: Innovative applications with artificial intelligence methods in neuroimaging data analysis

**DOI:** 10.3389/fnhum.2022.1108253

**Published:** 2022-12-16

**Authors:** Feng Liu, Li Zhao, Yuan-Chiao Lu, Yao Wu

**Affiliations:** ^1^Department of Radiology and Tianjin Key Laboratory of Functional Imaging, Tianjin Medical University General Hospital, Tianjin, China; ^2^Key Laboratory for Biomedical Engineering of Ministry of Education, College of Biomedical Engineering and Instrument Science, Zhejiang University, Hangzhou, China; ^3^Center for Neuroscience and Regenerative Medicine, Henry M. Jackson Foundation for the Advancement of Military Medicine, Bethesda, MD, United States; ^4^Developing Brain Institute, Children's National Hospital, Washington, DC, United States

**Keywords:** neuroimaging, medical image processing, artificial intelligence, computational modeling, brain disease diagnosis

Developing advanced analytic techniques to process neuroimaging data is crucial in advancing our understanding of the human brain structure and function. Most traditional image processing methods are unable to meet the accuracy and efficiency requirements of clinical practice and neuroscience research. Alternatively, advanced data processing methods such as artificial intelligence have yielded promising results in medical image analysis (Shen et al., 2017; England and Cheng, 2019), such as tumor detection (Saba et al., 2020; Sharif et al., 2020), brain registration (Wu et al., 2015; Fu et al., 2020; Wei et al., 2021), tissue segmentation (Wu et al., 2014; Ronneberger et al., 2015; Zhao et al., 2022), image reconstruction (Kainz et al., 2015; Cerrolaza et al., 2018), and neuropsychiatric disease diagnosis (Liu et al., 2015, 2017). Advanced artificial intelligence methods have shown improved accuracy and efficiency of neuroimaging data processing. As a result, this will advance the understanding of the human brain, which may assist in early diagnosis and developing intervention and/or surgery in patients with brain disorders. Currently, artificial intelligence is still in its infancy regarding its application to medical field and has the potential to be extensively used in clinical settings.

This Research Topic focuses on developing and applying artificial intelligence methods in medical image analysis, especially in the human brain, as well as using novel data processing methods and tools to address neuroimaging-related clinical and neuroscience questions. A total of 14 articles were exclusively selected and published in this topic.

## Brain structure segmentation

Two studies applied deep learning methods in brain structure segmentation using magnetic resonance imaging (MRI) scans. Theaud et al. proposed a DenseUNet-based deep learning segmentation algorithm for 10 tissues (i.e., white matter, gray matter, cerebrospinal fluid, ventricles, putamen, pallidum, hippocampus, caudate, amygdala, and thalamus) in diffusion weighted MR images. This method was trained and validated on 1,000 individuals from 22 to 90 years old from 5 public databases. Segmentation accuracy was superior to Freesurfer and FSL-FAST and the impacts on tractography were evaluated. Chai et al. proposed a contrast attention U-Net for deep gray matter nuclei segmentation in concatenated T1-weighted and quantitative susceptibility mapping sequences. This method was evaluated on two datasets acquired using different parameters from different MRI devices. Their results also suggested that sufficient data augmentation, deep supervision, and non-uniform patch sampling contributed to improving the segmentation accuracy.

## Brain functional images

Ren et al. employed Pearson's correlation and nearest neighbor to identify individuals in different conditions including right-handed tapping, left-handed tapping, foot tapping and resting state on functional near-infrared spectroscopy. Wang et al. proposed a functional MRI encoding model to study the hierarchy of neural auditory processing in the human brain through an unsupervised deep convolutional auto-encoder model. Their findings showed that the neural representation of hierarchical auditory features is not limited to the superior temporal gyrus, but is also related to the bilateral insula, ventral visual cortex, and thalamus. Yu et al. developed a computational framework that incorporates both spatial and temporal characteristics of the brain to investigate brain states and high-level semantic features from naturalistic functional MRI. The framework is shown to be effective in classifying audio categories and identifying semantically meaningful high-level features. Su et al. utilized support vector machine to identify brain activity changes in response to short-term real-world visual experience in a group of radiologists, which may provide novel insights into the neural mechanism of visual experts. Banerjee et al. proposed a seizure onset zone localization algorithm, namely “EPIK,” based on independent components derived from resting-state functional MRI in children with drug resistant epilepsy. EPIK outperforms support vector machine and convolutional neural network and shows consistent performance across different demographic subgroups.

## Feature extraction

An et al. proposed a semiautomatic prediction model for the rupture risk estimation of aneurysms, which consisted of multidimensional feature fusion, feature selection, and the construction of classification methods. Features included morphological features, radiomics features, clinical features, and deep learning features. Three dimensional EfficientNet-B0 was used to extract and analyze the classification capabilities of three sets of deep learning features (no-sigmoid features, sigmoid features, and binarization features). Five classification models were compared, and the *k*-nearest neighbor produced the best results. This study suggests that the full use of multidimensional feature fusion can improve the performance of aneurysm rupture risk assessment. Jiang et al. proposed a multi-scale feature extraction by the neural network with multi-task learning in continuous blood pressure estimation. Specifically, segmentation, denoising, and normalization were used to preprocess the target (electrocardiograph and photoplethysmography) and label signals (arterial blood pressure), and then a neural network with multi-task learning was designed to extract multi-scale features related to blood pressure from preprocessed target signals. Three blood pressure values (systolic blood pressure, diastolic blood pressure, and mean arterial pressure) were estimated simultaneously through multi-task learning, thus improving the accuracy of blood pressure estimation.

## Brain disease diagnosis

Liu et al. developed a method using decomposition-based correlation learning to capture the relationship between structural and functional MRI data. This method was evaluated in the classification of multiple neuropsychiatric disorders including schizophrenia, bipolar disorder, and attention deficit hyperactivity disorder. Chen et al. used MR spectroscopy to measure biochemical metabolites in prefrontal white matter and hippocampus in bipolar disorder patients with and without suicidal ideation, and combined brain biochemical metabolites with support vector machine algorithm to predict the severity of suicide risk in patients with bipolar disorder. Zhao, Han et al. proposed a hierarchical sub-network strategy to construct functional connectivity network from resting-state functional MRI based on matrix variate normal distribution theory. This method showed promising results in the classification of patients with autism spectrum disorder and normal controls. Zhao, Pan et al. proposed a scheme for constructing a high-order brain functional network from electroencephalography data based on sliding window, correlation, and clustering. Results demonstrate the efficiency of the high-order brain functional network in the identification of major depressive disorder. Hou et al. successfully employed linear support vector machine to classify patients with obstructive sleep apnea from healthy controls based on whole-brain resting-state functional connectivity, indicating these features can serve as neuroimaging biomarkers for this disorder.

The articles in this Research Topic proposed and applied advanced processing techniques in medical image analysis, mainly focusing on the human brain. This topic may benefit researchers and clinicians who are interested in artificial intelligence methods and neuroimaging data analysis.

## Author contributions

All authors listed have made a substantial, direct, and intellectual contribution to the work and approved it for publication.
